# A systematic review of international performance indicators and metrics relevant to UK general practice

**DOI:** 10.1136/bmjoq-2025-003477

**Published:** 2025-10-15

**Authors:** Duncan Chambers, Rebecca Mawson, Justina Mettle-Nunoo, Anthea Sutton, Andrew Booth

**Affiliations:** 1 Sheffield Centre for Health and Related Research (SCHARR), The University of Sheffield, Sheffield, England, UK; 2 School of Medicine and Population Health, The University of Sheffield, Sheffield, England, UK

**Keywords:** GENERAL PRACTICE, Quality improvement, Systematic Review

## Abstract

**Background:**

A wide variety of performance indicators/metrics are used to measure the performance of healthcare systems and to promote quality improvement (QI). We sought to identify indicators relevant to QI and organisational development (OD) within primary care/general practices and to evaluate the evidence for their use in QI and OD interventions in UK general practice and primary care.

**Methods:**

We used a framework based on UK National Health Service primary care documents to structure the review. Separate literature searches were performed in four databases to identify relevant reviews and primary studies. Studies were included if (1) the main focus was a metric or indicator that fell within the review framework or (2) they reported an OD or QI initiative or intervention in UK primary care that used one or more of the previously identified metrics or indicators. We mapped studies in group 1 against our framework domains. We performed a narrative synthesis of studies in group 2, again organised by the overall framework.

**Results:**

We included 28 studies, 24 (11 reviews and 13 international primary studies) for metrics or indicators and 4 for initiatives or interventions. The number of individual indicators or groups of indicators in group 1 studies ranged from 1 to 773. Three of the four UK QI/OD studies focused on initial access to general practice services; the other dealt with a programme to encourage self-care for long-term conditions. Mapping of the group 1 studies identified potentially relevant indicators across all domains but the process was methodologically challenging.

**Conclusions:**

Although numerous potential indicators exist, they tend to be poorly defined and lack examples of their use in practice. Further work is needed to identify and evaluate candidate indicators.

**WHAT IS ALREADY KNOWN ON THIS TOPIC**

**WHAT THIS STUDY ADDS**

- Our systematic review identified many studies of any indicators and groups of indicators that could be used to support internal (non-clinical) QI initiatives. However, we found limited evidence of such indicators being used in UK general practice and primary care.

**HOW THIS STUDY MIGHT AFFECT RESEARCH, PRACTICE OR POLICY**

- Our findings should prompt a discussion about the measures and metrics that really matter to different stakeholders, for example, policymakers, frontline primary care staff, patients, clinicians and researchers, and how to optimise their use in practice.

## Background

 In the UK National Health Service (NHS), general practitioners (GPs) and general practice staff provide primary medical care for acute and long-term conditions and refer patients for further investigation/treatment as required. A large percentage of the population is registered with a general practice and GPs treat patients throughout their lifespan. Primary care also covers community pharmacy, dental and optometry services. There are currently more than 6000 GP practices of varying sizes in England, and collectively, these now deliver over 360 million patient appointments per year (https://www.rcgp.org.uk/representing-you/key-statistics-insights#appointments).

Given the central role of general practice in the NHS, and increasing demand pressures on the workforce, it is important to collect and analyse data on the performance of the system and identify areas for improvement. Performance indicators have become increasingly prominent since the 1990s^1^ and the Quality and Outcomes Framework (QOF), introduced in 2004, linked general practice funding to the achievement of targets against a range of predominately clinical indicators. A 2015 review by the Health Foundation of general practice quality indicators in England^2^ argued that the publication of indicators provides transparency; supports informed choice and helps to empower patients and service users; supports accountability and performance management; and helps researchers to investigate workings of the healthcare system. A review of QOF was conducted in 2018 and recommended the inclusion of a quality improvement (QI) domain.^3^ A consultation on primary care incentives was launched in March 2024,^4^ although the results of this are not yet available, possibly because of the change of government in July 2024. Therefore, how to incentivise and measure quality improvement and performance in primary care remains a policy priority.

However, primary care performance is multidimensional, encompassing aspects such as clinical quality, access, patient experience, staff satisfaction, continuity of care, efficiency and equity. Despite the existence of various descriptive and analytical frameworks,^5^,^7^ capturing this complexity through a comprehensive set of indicators is challenging. While quantitative indicators like QOF targets and appointment availability provide valuable information, incorporating patient-reported outcome measures (PROMs) and patient-reported experience measures (PREMs) can offer a more holistic understanding of the quality of care from the patient’s perspective. While national indicators can facilitate benchmarking and comparisons between health providers, and used to target improvement efforts and interventions, it is equally important to allow for local adaptation and flexibility to address the specific population health needs, priorities of individual practices and requirements of local health systems and regions, including tackling health inequalities, as highlighted by an evaluation of the introduction of QI modules into the QOF from 2019.^8^ Involving different stakeholders, including healthcare professionals, patients and policymakers, in the development and implementation of performance indicators can further enhance their relevance, acceptance and usefulness.

While performance indicators can drive improvement, there is a risk of unintended consequences, such as narrowing the focus to only measurable aspects of care or encouraging gaming behaviours, especially when indicators are linked to financial payment and national targets. The availability of high-quality data and up-to-date information from across different parts of the health system is also key to successful implementation and use.

Difficulties experienced by patients attempting to access general practice services have been increasingly highlighted in recent years. Access can be measured quantitatively in terms of indicators like availability of same-day appointments or time to answer telephone calls. However, some researchers have argued that this approach fails to capture the real-world complexity of patients’ interactions with the healthcare system.^9^ Others have pointed out that general practices and primary care networks vary markedly in terms of contextual factors such as practice size, staffing levels, urban or rural location and social and economic deprivation.^8 10 11^ The presence of deprivation may lead to a practice’s performance appearing worse based on indicators such as the QOF or the measures used by the Care Quality Commission (CQC).^10^ Furthermore, appointment activity data does not fully capture the operational and business activity associated with running and managing a general practice, such as administrative tasks, digital transformation and handling ad hoc requests.

The current policy context in the UK NHS implies a need for improved measurement to support quality improvement initiatives in general practice. For example, NHS England’s General Practice Improvement Programme (GPIP), which started in 2023, offers tailored support to general practices and Primary Care Networks (PCNs, groups of linked general practices) to encourage improvement and change ways of working to help manage demand and align to a ‘modern general practice access’ model (https://www.england.nhs.uk/gp/national-general-practice-improvement-programme). We sought to identify and characterise relevant indicators from the international literature and to evaluate studies from the UK that involved QI/organisational development (OD) interventions or initiatives and measured impact on ‘real world’ service quality. An important aim of the review was to identify the types of measures available for improvements in non-clinical areas given a preponderance of measures traditionally focused on clinical outcomes in patients rather than practice processes.

## Methods

### Protocol and registration

The protocol was registered with PROSPERO (registration number CRD42023481064).

The full protocol is available via the funder’s website (https://fundingawards.nihr.ac.uk/award/NIHR164763). Variations from the protocol involved modifying the framework used for synthesis and using the study rather than the indicator as unit of analysis.

### Review questions

The review questions were as follows:

1. Which non-clinical metrics and indicators have been used to quantify service improvements in general practice and primary care internationally and which aspects of services do they address?
2. Which quality improvement or organisational development interventions/initiatives have been delivered in general practice and primary care in the UK to address these improvement domains?

### Eligibility criteria

Studies were included if (1) the main focus of the study was a metric or indicator of primary healthcare activity that corresponds to one or more of the domains of the framework described below (see ‘Synthesis methods’) and/or (2) they report an organisational development or quality improvement initiative or intervention that seeks to achieve change against one or more of the previously identified metrics or indicators. Detailed inclusion and exclusion criteria are presented in table 1.

**Table 1:** Inclusion and exclusion criteria

	Inclusion	Exclusion
Participants/population	General practice or primary healthcare staff and service users	Dental and community pharmacy staff and patients
Intervention/exposure	Metrics and indicators (qualitative or quantitative) of non-clinical quality improvement covered by the review framework (indicator groups containing clinical and non-clinical indicators were included) Quality improvement and organisational developments in UK general practice and other primary care settings addressing any of the above domains	Metrics and indicators specific to a specific disease or condition; metrics of capability building (ie, data-driven improvement, culture, leadership, governance, digital capabilities)
Comparator(s)	Metrics and indicators from high-income countries with comparable primary care/general practice systems to the UK (Australia, Canada, Ireland, New Zealand, Scandinavia and Netherlands). Comparative and non-comparative studies	Individual metrics/indicators developed in the USA
Outcomes 1.	Feasibility and usefulness of the metric or indicator; external validity; face validity; ease of interpretation; limitations of the metric/indicator; staff satisfaction with metrics and indicators; patient/carer acceptance of the metric or indicator	
Outcomes 2.	Facility to address specific metrics or indicators Effect size or proportion of change achieved Patient acceptability of the initiative/intervention Staff acceptance of the initiative/intervention Mitigation of inequalities through the intervention/initiative Feasibility of providing data collected systematically and regularly and used to inform improvements Additional outcomes: details of development of the metric or indicator; process data on implementation; resource costs/time/time savings (if present)	
Study type	Any study type Studies must report research (quantitative or qualitative; from research, evaluation or audit)	
Other	Peer-reviewed published research or substantive ‘grey literature’ reports	Studies focusing on low-income and middle-income countries Conference abstracts, theses or dissertations

### Information sources

Two searches were conducted: (1) to identify systematic reviews and (2) to identify primary research. Searches for systematic reviews were conducted on Ovid MEDLINE, Embase via Ovid, HMIC (Health Management Information Consortium database) via Ovid, Scopus, Science and Social Sciences Citation Indexes via Web of Science. Due to the rapid nature of the review, a condensed number of information sources were prioritised to identify primary research; Ovid MEDLINE, Embase via Ovid, Science and Social Sciences Citation Indexes via Web of Science. Grey literature was also searched, specifically for primary research.

### Search strategy

#### Systematic reviews search (Search 1)

The search for systematic reviews aimed to map the metrics and indicators used to quantify service improvements in general practice and primary care. It was conducted between 27 October and 28 November 2023. Search terms (subject headings and free-text) were grouped according to three concepts: general practice/primary care setting, metrics and indicators, and NHS England’s GPIP domains. A methodological search filter to identify systematic reviews was combined with the topic search terms. No search limits were applied.

#### Primary research studies search (Search 2)

The second search aimed to identify primary research on quality improvement or organisational development interventions/initiatives delivered in general practice and primary care in the UK to address the GPIP improvement domains. It was conducted between 28 November and 18 December 2023. Search terms (subject headings and free-text) were grouped according to three concepts: general practice/primary care setting, metrics and indicators, and NHS England’s GPIP domains. A geographical search filter to identify studies conducted in the UK was combined with the topic search terms. Searches were limited to 2013 onwards. The following websites were searched for grey literature: The Health Foundation, NHS England and The Strategy Unit. The TRIP (Turning Research into Practice) database was searched, along with the preprint server medRxiv. The search engines Google and Google Scholar were also searched using terms relating to metrics, indicators and general practice.

Sample search strategies are presented in Appendix 1 and the full set of search strategies is available as online supplemental file 1.

### Study selection process

Study selection at the title and abstract stage involved dual independent selection by two reviewers from 10% of the records. Following confirmation of an acceptable level of consistency, remaining records were screened by a single reviewer. At full-text screening, studies were assessed against the inclusion criteria by two reviewers acting independently; disagreements were resolved by consensus among the review team. Included studies were filtered into those reporting the indicator/metric and those that reported use of a metric/indicator within a primary care quality improvement or organisational development initiative/intervention.

### Data collection process

For the review of metrics/indicators, our initial aim was to extract data using the metric/indicator as the unit of analysis, supported by all identified publications relevant to that metric or indicator. However, a higher than anticipated number of indicators meant that it was only feasible to extract data using the study as the primary unit of analysis. Available data on individual indicators were extracted from primary research studies providing relevant information. We also extracted data on relevant indicators (ie, excluding condition-specific indicators) from the supplementary file published with the umbrella review by Ramalho *et al*.^12^ Interventions/initiatives were analysed by each study describing that individual initiative/intervention.

Data were extracted by one reviewer and checked by a second reviewer using prespecified forms in Microsoft Excel or Word tailored to the amount and type of data to be extracted.

### Data items

For review question 1, we extracted data on study design and methodology, metric/indicator name, characteristics, organisational context (eg, urban/rural, practice size (patient list), workforce, practice-level Index of Multiple Deprivation (IMD)), validation, together with any measures of effect (where applicable).

Data items for review question 2 were study characteristics, setting, intervention components, metrics used and effect size or change achieved.

We also extracted data where possible on the prespecified outcomes for each group of studies (see table 1 for list of outcomes).

### Risk of bias assessment

There is no formal method for assessing studies reporting metrics and indicators. Instead, we focused on the individual metrics or indicators themselves, assessing information from any studies that contributed to an understanding of that metric or indicator. Specifically, we based our assessments on the Agency for Healthcare Research and Quality (AHRQ) quality indicator properties (https://www.ahrq.gov/talkingquality/measures/measure-questions.html). The AHRQ criteria measure standardisation, comparability across organisations, data availability, timeliness, relevance to target audience, validity, experience, stability, evaluability, distinguishability and credibility.

Studies of QI/OD interventions were not formally assessed for risk of bias because our focus was on the role of indicators/metrics. Where data allowed, organisational development and quality improvement interventions and initiatives were evaluated according to the following predefined measures of intervention effectiveness:

- Facility to address specific metrics or indicators.
- Effect size or proportion of change achieved.
- Patient acceptability of the initiative/intervention.
- Staff acceptance of the initiative/intervention.
- Mitigation of inequalities through the intervention/initiative.
- Feasibility of providing data collected systematically and regularly and used to inform improvements (ie, quality control and assurance, eg, quality scorecards, dashboards, clinical audit, patient surveys).

### Synthesis methods

We conducted a narrative synthesis grouping studies by the two review questions. Studies relevant to question 1 were used to develop a ‘map’ of available indicators. Studies relevant to question 2 were used to evaluate the quantity and quality of UK-based QI/OD interventions. For both syntheses, we grouped studies using a framework based on the GPIP practice level support framework (https://www.england.nhs.uk/gp/national-general-practice-improvement-programme/support-level-framework) validated by reference to key NHS primary care documents. The framework had six domains: access to primary care systems, access to primary care clinical services, care navigation and triage, managing demand and capacity, managing the practice workload, and an additional category covering key objectives including time and cost savings and improvements in patient experience.

We anticipated that features of the metrics and indicators, and associated interventions, would be heterogeneous. We planned to investigate this heterogeneity using (1) the outcomes presented in table 1 and (2) the metric/indicator properties used to assess metric/indicator quality, and to present synthesised data in the form of tables, narrative summaries and diagrams as appropriate.

### Public involvement

We discussed the project with members of the standing public advisory group for the Sheffield HS&DR Evidence Synthesis Centre. We used the domains listed above as a focus for the meeting to identify the aspects of most importance to patients. We also asked the group for suggestions in relation to the evaluation of QI/OD programmes in general practice.

Three members of the public advisory group of diverse age, sex/gender and ethnicity provided feedback. Members identified access to primary care systems as being particularly important, with continued availability of face-to-face appointments seen as a key metric. Participants stressed the importance of requesting feedback from patients by regular surveys as a means of evaluating initiatives to improve quality of general practice and other primary care services. Participants also emphasised the dramatic changes that have taken place in general practice following the COVID-19 pandemic and suggested that a distinction between research from before and after the pandemic would be helpful in evaluating the relevance of the indicators used.

## Results

### Results of literature search

**Figure 1:**
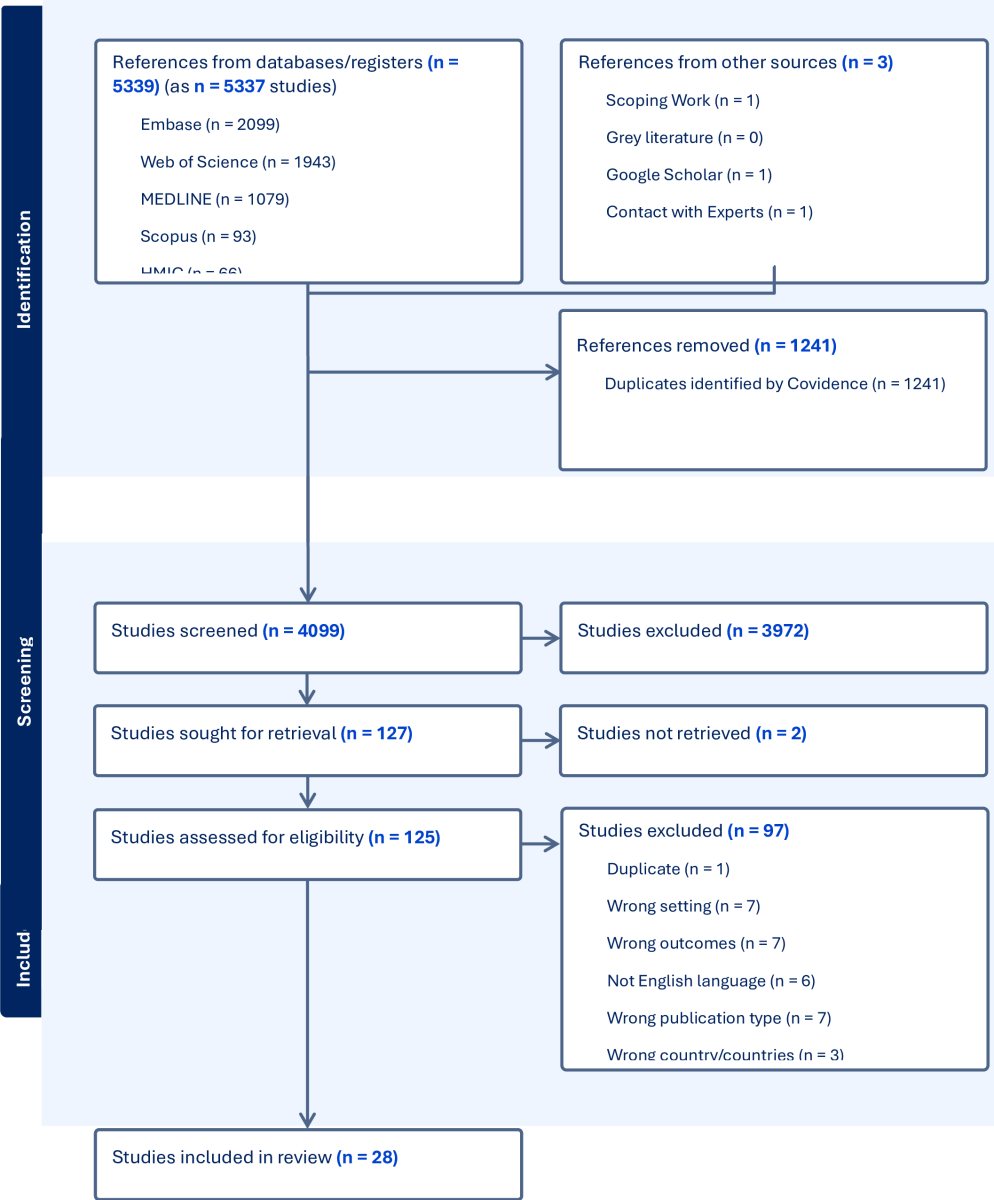
PRISMA 2020 flow diagram. HMIC, Health Management Information Consortium.

The results of the literature search and screening process are summarised in the Preferred Reporting Items for Systematic Reviews and Meta-Analyses (PRISMA) 2020 flow diagram (figure 1).

We included a total of 28 studies (30 publications: 28 peer-reviewed and 2 grey literature reports), of which 24 addressed review question 1 (mapping of available indicators) and 4 (6 publications) addressed question 2 (use of indicators in UK QI/OD interventions and initiatives).

### Mapping of relevant metrics/indicators

#### Map of studies

The map of available indicators drew on data from 11 systematic and non-systematic reviews (including one umbrella review^12^ and 13 primary research studies). The number of individual indicators or groups of indicators covered ranged from 1 to 773. Studies varied widely in scope and methodology and covered diverse topics related to measuring and improving quality in primary care^13^,^15^ ^16^ 17,20 (see online supplemental file 2).

#### Map of indicators

Indicators included in the umbrella review by Ramalho *et al*^12^ are summarised in online supplemental file 3. Of 727 indicators included in the umbrella review, 15 mapped to our framework domains 1a and 1b, 7 to domain 2, 6 to domain 3, 1 to domain 4 and 8 to domain 5. A considerable majority of indicators measured clinical processes such as prescribing and monitoring of long-term conditions.

#### Summary description of indicators

Most included studies provided descriptions of indicators in terms of how they are calculated in the text (eg, Alsabbagh 2020^21^), in supplementary files or by reference to earlier papers. However, the predefined ‘indicator outcomes’ for this review were less commonly reported. It should also be borne in mind that most of the indicators and metrics included in reviews that addressed our review question 1 covered clinical aspects of general practice and primary care outside the scope of the review. For these reasons, we extracted ‘indicator outcome’ data from four studies^22^,^25^ (table 2).

**Table 2:** Summary of ‘indicator outcomes’

	Indicator and framework domain(s)* covered	Feasibility and usefulness	External validity	Face validity	Ease of interpretation	Limitations	Staff satisfaction	Patient/carer acceptance
Crossland 2014^22^	Primary Care Practice Improvement Tool (PC-PIT) Domain 2	19/28 participants rated PC-PIT as useful	Participants emphasised the relevance of the PC-PIT to everyday practice work and planning	19/28 said PC-PIT captured key elements of practice organisation and function	Required a high reading age; many administrative and reception staff found it difficult to understand	Some simplification required	Paper reports pilot study Administrative staff less satisfied than GPs	NR
Howie 2000^23^	Consultation Quality Index (CQI) Domain 5	Considered useful for assessing quality of care by doctors and practices; requires patients to complete a questionnaire	Results were independent of practice case mix and level of deprivation	Authors state results had strong face validity	Divided into bands 1 (least good) to 6 (best) so relatively easy to interpret	Seen as one of a range of quality measures	NR	NR but definition of need and assessment of outcome were both determined exclusively by patient responses.
Levesque 2012^24^	Group of 17 validated tools Domains 1b; 2; 5	Provides data to assess potential usefulness	Tools had undergone different levels of quantitative and qualitative validation		NR	Important attributes of primary care were not covered by any of the validated tools	NR	NR but all instruments aimed to evaluate quality from the patient perspective
Sidaway-Lee 2019^25^	St Leonard’s Index of Continuity of Care (SLICC) Domain 5	Described as simple to calculate and useful because continuity of care has benefits for patients	NR but includes all appointments	NR but comparison with UPC (Usual Provider of Care) index provided	High (percentage of appointments with patient’s personal doctor)	Study involved one practice where all GPs were part-time	NR but authors note that GPs have incentive to record appointments accurately for workload monitoring	NR

* 1a, access to primary care systems; 1b, access to primary care clinical services; 2, care navigation and triage; 3, managing demand and capacity; 4, managing the practice workload; 5, key outcome objectives.

GPs, general practitioners; NR, not reported.

### Quality of indicators

As noted above, the study (covering one or multiple indicators) rather than the indicator (using information from multiple studies where available) was designated as the preferred unit of analysis. Ten primary studies reporting on indicators or groups of indicators were assessed against the AHRQ criteria (online supplemental file 4). While data were lacking for some items, most indicators were assessed positively for relevance, validity, evaluability and credibility.

### Use of relevant metrics/indicators in UK QI/OD interventions or initiatives

#### Study characteristics

The four included UK quality improvement studies (table 3) evaluated four diverse interventions aimed at improving general practice processes: online consultations,^26^ GP-led or nurse-led telephone triage,^27^ promotion of self-care^28^ and use of shorter prebookable review appointments for patients needing follow-up after an initial appointment.^29^ Three of the studies had a control group and one was a cluster randomised controlled trial.^27^

**Table 3:** Summary of UK quality improvement intervention studies

Study reference	Study design	Setting	Details of QI intervention or initiative	Metric(s) or indicator(s) used	Results (effect size or change achieved, relevant to GPIP domains)
Carter 2018^26^	Mixed method study	England, UK	Intervention: webGP (online consultation) Comparator: face-to-face consultations	Volume of consultations Patient surveys Staff surveys Administrative staff workload	No impact on practice workload 72% of webGP requests required face-to-face or telephone consultation Good survey responses from patients regarding timeliness and quality of care (low response rate—35.1%) Partial acceptance of intervention by staff with concerns of negative interactions with pre-existing practice systems.
Murdoch 2015^27^	Cluster randomised controlled trial	England, UK	Intervention: GP-/nurse-led telephone triage Comparator: usual care	Patient safety Patient satisfaction Healthcare costs Patient-provider communication Staff surveys	28% reduction in patient-GP contact time 31% reduction in GP face-to-face contacts, and a relative reduction of 1.4 min in overall GP contact time Nurse triage costs equivalent to both GP triage and usual care
Robertson 2013^28^	Mixed method study	England, UK	Intervention: Supporting Self-Care in General Practice programme	GP appointment time duration Telephone consultation Online consultations Patient-centred care	NR (mainly qualitative data reported)
Slater 2021^29^	Mixed method study	Scotland, UK	Intervention: shorter prebookable review appointments Comparator: ‘On the day’ review appointments	Patient accessibility to care Number of GP appointments per day Efficiency of appointment system Number of calls per day Continuity of care Administrative staff workload	43% increase in available appointments (554 to 792 appointments per 2-week period) Increased number of patients seen at the GP Median 93% same day appointments Administrative staff workload remained the same in terms of number of phone calls received per day Administrative staff workload reduced in terms of dealing with frustrated patients Improved staff survey results Increased rate of DNAs (did not attend) with appointments prebooked

GP, general practitioner; GPIP, General Practice Improvement Programme; QI, quality improvement.

Indicators used in the included studies (table 3) mainly focused on measures of access (domains 1a and 1b, for example, numbers of face-to-face, online and telephone appointments; consultation length; number of same-day appointments) and practice workload (domain 4, primarily administrative staff workload^26 29^). The study of self-care for people with long-term conditions^28^ differed from the others in presenting a mainly qualitative summary of findings (supported by relevant quotations), although it was clear from the description of the programme that quantitative data were also collected.

Additional QI work to support implementation of the study interventions was reported in three studies.^27^,^29^

#### Intervention effectiveness

Interventions (including both the primary intervention and supporting QI work) showed no clear pattern of effects (in terms of magnitude or direction) on the reported indicators (table 3). The indicators used were generally appropriate for the intervention being evaluated and quantitative measures of change were captured in three studies.^26 27 29^ Patient and staff acceptability was addressed in three studies,^26 27 29^ with additional measures of workload in two studies.^26 29^ None of the included studies reported explicitly on mitigation of inequalities by the intervention. However, introduction of electronic consultations^26^ may risk increasing inequality in access at the expense of patients without access to digital technology. The other reported interventions could potentially reduce inequality by matching appointments to need^27 29^ and by prioritising a vulnerable population of patients with long-term conditions.^28^ All four of the included studies included data that could be collected systematically and used to inform further improvements.

#### Synthesis across evidence sources

Online supplemental file 5 summarises the relationship between indicators mapped from the literature and those used in UK studies. For domains 1a and 1b, focusing on access, the UK QI studies used a limited number of measures, primarily related to number, type (face-to-face vs online) and duration of consultations. The Ramalho umbrella review identified other potentially useful indicators (eg, use of urgent appointments; practice opening hours; out-of-hours services; and home visits). There was little overlap between the umbrella review and UK studies. Metrics and indicators related to telephone access were not present in the umbrella review and were only present in one of the UK studies.^29^

With respect to care navigation and triage (domain 2), the UK studies gathered data from patient surveys. Ramalho *et al* also included indicators of patient compliance with advice received which could reflect quality of triage by general practice staff. Other indicators mentioned in the umbrella review were less clearly defined, for example, ‘Differentiates appropriately between important and minor issues’ and ‘Does not raise patient expectations that recommendations will be implemented’.

The QI study by Slater *et al*^29^ corresponded with managing demand and capacity (domain 3) as it aimed to implement and evaluate a more efficient appointment system. Ramalho *et al* mentioned some other indicators around this domain, but again, limited detail was available.

In terms of managing the whole practice workload (domain 4), two UK QI studies used administrative staff workload as an indicator.^28 29^ A similar indicator (‘Development of the primary care workforce’) was identified by Ramalho *et al.* The four questionnaires described by Schafer *et al*^30^ included questions potentially relevant to this domain, although these were split between different questionnaires.

The final framework domain (domain 5) covers diverse key QI outcomes, such as improved patient experience. In the UK QI studies, this domain was represented by staff surveys in three studies.^31^,^33^ Ramalho *et al* identified other indicators in this domain, for example, around record keeping (online supplemental file 5) and our mapping review included several relevant studies, particularly the recent work of Benson *et al*^34 35^ and the older European Practice Assessment instrument.^31 36^

## Discussion

### Main findings

We identified large numbers of indicators/indicator groups but few were relevant to our review focus on indicators of improvement in practice processes; most measured clinical aspects of primary care such as treatment of specific conditions, monitoring of long-term conditions or prescribing. Indicators often appeared to be designed for academic research rather than for pragmatic general practice, that is, to measure effects of interventions in studies rather than supporting front line improvement.

The UK QI/OD literature was very limited, in contrast to plentiful examples of initiatives and interventions focusing on clinical indicators. The four included studies covered a diverse group of interventions and supporting QI work. Three of the studies focused on initial access to GPs’ services,^26 27 29^ reflecting the distribution of indicators seen in the wider literature (online supplemental file 5) and the views expressed by our patient/public involvement (PPI) group.

The balance of the literature was skewed towards reviews and descriptions of groups of indicators, with relatively few studies focusing on a single metric or indicator. This meant that reporting of indicator properties was varied and made it difficult for us to use the indicator as the unit of analysis as originally planned. The process of identifying indicators at different levels of literature (umbrella reviews, reviews and primary studies) was challenging. The umbrella review by Ramalho and colleagues was helpful but most of the included reviews were not specific to non-clinical QI in primary care or general practice.

The indicators and metrics identified could be broadly grouped into those intended for internal use within practices (eg, to support ongoing quality improvement work) and those intended for higher level decision-makers and/or patients and the public. Single measures tended to fall into the first category^23 25^ while decision-makers and the public were offered diverse groups of measures, sometimes identified as ‘monitors’ or ‘dashboards’.^37 38^ None of the included groups of indicators corresponded closely with domains of the framework used for this review or focused mainly on non-clinical aspects of general practice. This suggests that evaluation of large-scale QI initiatives would require purposive selection among existing indicators and/or development of new ones.

### Strengths and limitations

#### Strengths

The review has a number of strengths. We used a dual approach to map the available indicators against those that have been used in practice in the UK. Identification and re-analysis of a subset of relevant indicators among the large number included in an umbrella review increased the yield and added value by increasing the relevance of the review to QI in UK general practice. We built on standard systematic review processes by use of novel methods to characterise indicators/metrics per se and in relation to their role in QI interventions/initiatives.

#### Limitations

We acknowledge the large and diverse literature covering QI in primary care (and healthcare generally) which touches on different dimensions of improvement. It is likely that a large number of indicators used in the literature are clinical and have been driven by policy levers (eg, QOF and CQUIN (Commissioning for Quality and Innovation)) and clinical academic research. Indicators related to team climate and culture^39^ were excluded at the protocol stage to manage the size of the review and could be an area for future review work. Use of the study rather than the indicator as the unit of analysis limited our ability to assess indicator quality and should be taken into account in interpreting the findings. The limited coverage of this review reflects the prespecified review questions and the limited number of QI/OD interventions/initiatives identified from the UK. Finally, although we endeavoured to exclude individual indicators from the USA, the international literature was inevitably dominated by evidence from primary care systems very different from those of the UK nations. This should be borne in mind when evaluating indicators and associated incentives derived from social insurance-based (like most of Europe) or fee for service-based (common in the USA) primary care models.

### Implications for decision makers

For policymakers, commissioners and practitioners, further work is needed to cover operational and other improvement domains in primary care and associated metrics. Our findings suggest that there may be a need for a discussion about the measures and metrics that really matter to different stakeholders, for example, policymakers, frontline primary care staff, patients, clinicians and researchers. It appears that there could be a disconnect between the academic literature and QI metrics reported and what is actually needed to track quality improvement and transformation/change over time in primary care practice, in a way that is meaningful to patients and staff. The increasing emphasis in UK practice on delivery of QI through PCNs, and increasing co-operation and co-location of GPs with other health professionals, also support the need to rethink how QI is delivered, measured and reported. Our findings also support the need for caution when using indicators to compare practices serving different population groups, which can penalise practices in more deprived areas (by around 7% when weighted for need in one recent study^40^).

Situations also arise where indicators may conflict, for example, staff satisfaction and patient satisfaction. Happy staff in a practice with clear boundaries around access might mean unhappy patients who see themselves as denied the access they want. The challenge for decision-makers is to find a set of measures to drive improvement efforts (as opposed to performance management) which give an accurate picture of what modern day practice life and activity looks like, from the view of patients and staff; for example, unlimited patient access to a GP is not an achievable aim. A report from the National Institute for Health Research Collaboration for Leadership in Applied Health Research and Care (NIHR CLAHRC) South West Peninsula (excluded from the review because of lack of empirical data) examined the SERVQUAL approach, which measures service quality using self-report.^41^ This approach views quality as a subjective concept, making it amenable to use with quality impact assessment.

The National Voices patient organisation published ‘A vision for the future of primary care’ in June 2023, identifying nine priority areas for improvement.^42^ We initially considered a broad agenda such as this as the framework for our review but subsequently agreed to focus on a more limited group of domains. Our approach to mapping indicators and metrics against studies could potentially be adapted and extended to cover additional aspects of primary care and general practice.

## Conclusions

We identified large numbers of indicators/indicator groups but few were relevant to our review questions. The UK QI/OD literature was very limited, although the four included studies covered a diverse group of interventions and supporting QI work. Findings of our mapping review suggest that a large number of indicators used in the literature are clinical and have been driven by policy levers (eg, QOF and CQUIN (Commissioning for Quality and Innovation)) and clinical academic research. Further work is needed to identify and evaluate candidate indicators from the dispersed literature and to optimise their value for patients, practitioners and decision-makers concerned with measuring quality in general practice and primary care.

## Supplementary material

10.1136/bmjoq-2025-003477 online supplemental file 1

10.1136/bmjoq-2025-003477 online supplemental file 2

10.1136/bmjoq-2025-003477 online supplemental file 3

10.1136/bmjoq-2025-003477 online supplemental file 4

10.1136/bmjoq-2025-003477 online supplemental file 5

## Data Availability

All data relevant to the study are included in the article or uploaded as supplementary information.
