# Supplementary material for: A systematic review of international performance indicators and metrics relevant to UK general practice
Source: BMJ Open Qual. 2025 Oct 15;14(4):e003477. doi: 10.1136/bmjoq-2025-003477 (PMC12530429; doi:10.1136/bmjoq-2025-003477)
Supplement: online supplemental file 1 [file bmjoq-14-4-s001.docx]

**Supplementary file 1: Search strategies**

**Search 1: Systematic Reviews**

**Ovid MEDLINE(R) and Epub Ahead of Print, In-Process, In-Data-Review & Other Non-Indexed Citations, Daily and Versions <1946 to October 26, 2023>**

1 *Primary Health Care/ 55560

2 (primary adj3 care).tw. 173956

3 (pc or pcn*).tw. 110682

4 *General Practice/ 12598

5 "general practice".tw. 38817

6 gp.tw. 49612

7 or/1-6 369425

8 *Benchmarking/ 7237

9 "indicator*".tw. 370503

10 metrics.tw. 71178

11 *"Weights and Measures"/ 1396

12 measurements.tw. 837166

13 measures.tw. 939608

14 indices.tw. 192717

15 Quality Assurance, Health Care/ or *Quality Indicators, Health Care/ 65519

16 or/8-15 2310350

17 "national general practice improvement programme*".tw. 0

18 gpip.tw. 59

19 "national voice*".tw. 40

20 *health services accessibility/ or *access to primary care/ 42978

21 ((patient* or telephone* or online* or virtual*) adj3 (access* or consult* or visit* or appointment*)).tw. 88402

22 *Triage/ 7842

23 "triag*".tw. 28297

24 "care navigation*".tw. 153

25 *Workload/ 9957

26 (manag* adj3 workload*).tw. 819

27 "standard actions and outcomes".tw. 0

28 ((time or cost) adj3 sav*).tw. 42765

29 *"Cost Savings"/ 2249

30 *Patient Satisfaction/ 32139

31 "patient experience*".tw. 24031

32 *patient-centered care/ or *patient navigation/ 14616

33 person-centred care.tw. 1908

34 (staff adj3 (morale or condition* or experience*)).tw. 5118

35 "access to care".kw. 2499

36 "telephone access".kw. 1

37 *"Appointments and Schedules"/ 4997

38 ((prioritis* or allocat*) adj2 (care or service* or resource*)).tw. 22189

39 ((multi-professional or multi professional) adj (team* or framework*)).tw. 449

40 optimi?ation.tw. 203038

41 (information-gathering or information gathering).tw. 1356

42 (matching adj5 needs).tw. 391

43 expressed need*.tw. 559

44 ((episodic or continuity) adj2 care).tw. 9970

45 (integrated adj1 working).tw. 125

46 *Quality Improvement/ 16414

47 (quality improvement or QI).tw. 55273

48 or/17-47 574003

49 (indicator* or metrics or measurements or measures or indices).ti. 217485

50 7 and 49 2761

51 7 and 16 and 48 7224

52 50 or 51 9584

53 (MEDLINE or systematic review).tw. or meta analysis.pt. 441603

54 52 and 53 287

**Embase <1974 to 2023 Week 45>**

1 *primary health care/ 35362

2 (primary adj3 care).tw. 237278

3 (pc or pcn*).tw. 160405

4 *general practice/ 40273

5 "general practice".tw. 45013

6 gp.tw. 74475

7 or/1-6 519553

8 *benchmarking/ 3135

9 "indicator*".tw. 481266

10 metrics.tw. 100007

11 *standard/ 5189

12 measurements.tw. 1004621

13 measures.tw. 1180913

14 indices.tw. 243469

15 health care quality/ 276869

16 *quality control/ 37201

17 or/8-16 3076200

18 "national general practice improvement programme*".tw. 0

19 gpip.tw. 69

20 "national voice*".tw. 50

21 *health care access/ 13956

22 *primary care access/ 39

23 ((patient* or telephone* or online* or virtual*) adj3 (access* or consult* or visit* or appointment*)).tw. 158496

24 *patient triage/ 1301

25 "triag*".tw. 44852

26 "care navigation*".tw. 248

27 *workload/ 12185

28 (manag* adj3 workload*).tw. 1132

29 "standard actions and outcomes".tw. 0

30 ((time or cost) adj3 sav*).tw. 66941

31 *"cost control"/ 9270

32 *patient satisfaction/ 29220

33 "patient experience*".tw. 41283

34 *patient care/ 81809

35 person-centred care.tw. 2326

36 (staff adj3 (morale or condition* or experience*)).tw. 7481

37 "access to care".kw. 2827

38 "telephone access".kw. 1

39 *hospital management/ 28264

40 ((prioritis* or allocat*) adj2 (care or service* or resource*)).tw. 27465

41 ((multi-professional or multi professional) adj (team* or framework*)).tw. 792

42 optimi?ation.tw. 252759

43 (information-gathering or information gathering).tw. 1770

44 (matching adj5 needs).tw. 526

45 expressed need*.tw. 778

46 ((episodic or continuity) adj2 care).tw. 13685

47 (integrated adj1 working).tw. 194

48 *total quality management/ 29549

49 (quality improvement or QI).tw. 90166

50 or/18-49 849114

51 (indicator* or metrics or measurements or measures or indices).ti. 249707

52 7 and 51 3848

53 7 and 17 and 50 12028

54 52 or 53 15314

55 meta-analys:.mp. or search:.tw. or review.pt. 3882997

56 54 and 55 1348

57 limit 56 to (embase or medline or "preprints (unpublished, non-peer reviewed)") 1183

**Cochrane Database of Systematic Reviews**

#1 MeSH descriptor: [Primary Health Care] this term only 5833

#2 (primary NEAR/3 care):ti 9013

#3 (pc or pcn*):ti 1001

#4 MeSH descriptor: [General Practice] this term only 827

#5 "general practice":ti 2231

#6 gp:ti 547

#7 {OR #1-#6} 15032

#8 MeSH descriptor: [Benchmarking] this term only 230

#9 indicator* 26234

#10 metrics 4565

#11 MeSH descriptor: [Weights and Measures] this term only 45

#12 measurements 83348

#13 measures 184767

#14 indices 216895

#15 MeSH descriptor: [Quality Assurance, Health Care] explode all trees 5797

#16 MeSH descriptor: [Quality Indicators, Health Care] this term only 292

#17 {OR #8-#16} 440881

#18 national general practice improvement programme* 2271

#19 gpip 12

#20 national voice* 780

#21 MeSH descriptor: [Health Services Accessibility] this term only 982

#22 MeSH descriptor: [Access to Primary Care] this term only 0

#23 ((patient* or telephone* or online* or virtual*) NEAR/3 (access* or consult* or visit* or appointment*)) 22497

#24 MeSH descriptor: [Triage] this term only 408

#25 triag* 2538

#26 care navigation* 1283

#27 MeSH descriptor: [Workload] this term only 714

#28 (manag* NEAR/3 workload*) 78

#29 "standard actions and outcomes" 0

#30 ((time or cost) NEAR/3 sav*) 5886

#31 MeSH descriptor: [Cost Savings] this term only 539

#32 MeSH descriptor: [Patient Satisfaction] this term only 14800

#33 patient experience* 57386

#34 MeSH descriptor: [Patient-Centered Care] this term only 850

#35 MeSH descriptor: [Patient Navigation] this term only 193

#36 person-centred care 1020

#37 (staff NEAR/3 (morale or condition* or experience*)) 711

#38 "access to care" 921

#39 "telephone access" 92

#40 MeSH descriptor: [Appointments and Schedules] this term only 543

#41 ((prioritis* or allocat*) NEAR/2 (care or service* or resource*)) 1800

#42 ((multi-professional or multi professional) NEXT (team* or framework*)) 155

#43 optimi?ation 7737

#44 (information-gathering or information gathering) 1033

#45 (matching NEAR/5 needs) 29

#46 expressed need* 8778

#47 ((episodic or continuity) NEAR/2 care) 874

#48 (integrated NEAR/1 working) 7

#49 MeSH descriptor: [Quality Improvement] this term only 1023

#50 (quality improvement or QI) 54174

#51 {OR #8-#50} 539866

#52 ((indicator* or metrics or measurements or measures or indices)):ti (Word variations have been searched) 30714

#53 #7 AND #52 191

#54 #7 AND #17 AND #51 4914

#55 #53 OR #54 7194

**Scopus**

( ( TITLE-ABS-KEY ( ( indicator* OR metrics OR measurements OR measures OR indices ) ) ) AND ( TITLE-ABS-KEY ( ( primary AND care OR primary AND health AND care OR primary AND healthcare OR general AND practice OR gp ) ) ) ) AND ( ( TITLE-ABS-KEY ( ( "national general practice improvement programme*" OR gpip OR "national voice*" OR triag* OR "care navigation*" OR "standard actions and outcomes" OR "patient experience*" OR person-centred AND care OR "access to care" OR "telephone access" OR optim?ation OR information-gathering OR information AND gathering OR expressed AND need* OR quality AND improvement OR qi ) ) ) OR ( TITLE-ABS-KEY ( ( ( patient* OR telephone* OR online* OR virtual* ) W/3 ( access* OR consult* OR visit* OR appointment* ) ) ) ) OR ( TITLE-ABS-KEY ( ( manag* W/3 workload* ) ) ) OR ( TITLE-ABS-KEY ( "standard actions and outcomes" ) ) OR ( TITLE-ABS-KEY ( ( ( time OR cost ) W/3 sav* ) ) ) OR ( TITLE-ABS-KEY ( ( staff W/3 ( morale OR condition* OR experience* ) ) ) ) OR ( TITLE-ABS-KEY ( ( ( prioritis* OR allocat* ) W/2 ( care OR service* OR resource* ) ) ) ) OR ( TITLE-ABS-KEY ( ( ( multi-professional OR "multi professional" ) W/0 ( team* OR framework* ) ) ) ) OR ( TITLE-ABS-KEY ( ( matching W/5 needs ) ) ) OR ( TITLE-ABS-KEY ( ( ( episodic OR continuity ) W/2 care ) ) ) OR ( TITLE-ABS-KEY ( ( integrated W/1 working ) ) ) ) ) AND ( TITLE ( review OR reviews OR meta-analysis OR meta-analyses ) )

**HMIC Health Management Information Consortium <1979 to September 2023>**

1 primary care/ 12757

2 (primary adj3 care).tw. 21183

3 (pc or pcn*).tw. 278

4 General practice/ 8903

5 "general practice".tw. 8407

6 gp.tw. 7983

7 or/1-6 36374

8 Benchmarking/ 532

9 "indicator*".tw. 6689

10 metrics.tw. 304

11 measurements.tw. 1650

12 measures.tw. 17550

13 indices.tw. 854

14 "Quality of patient care"/ or Quality improvement/ or Quality assurance in health services/ or Quality assurance/ or Standards/ 21054

15 or/8-14 43812

16 "national general practice improvement programme*".tw. 0

17 gpip.tw. 0

18 "national voice*".tw. 39

19 Access to health services/ 7618

20 ((patient* or telephone* or online* or virtual*) adj3 (access* or consult* or visit* or appointment*)).tw. 3916

21 Triage/ 307

22 "triag*".tw. 675

23 "care navigation*".tw. 4

24 Workload/ 1369

25 (manag* adj3 workload*).tw. 124

26 "standard actions and outcomes".tw. 0

27 ((time or cost) adj3 sav*).tw. 1243

28 Economies/ 437

29 Patient satisfaction/ 2905

30 "patient experience*".tw. 1131

31 patient centred care/ 1521

32 patient experience/ 1614

33 person-centred care.tw. 233

34 (staff adj3 (morale or condition* or experience*)).tw. 1039

35 "telephone access".tw. 33

36 Patient appointments/ or Scheduling/ 320

37 ((prioritis* or allocat*) adj2 (care or service* or resource*)).tw. 2306

38 ((multi-professional or multi professional) adj (team* or framework*)).tw. 42

39 optimi?ation.tw. 252

40 (information-gathering or information gathering).tw. 136

41 (matching adj5 needs).tw. 43

42 expressed need*.tw. 70

43 ((episodic or continuity) adj2 care).tw. 1080

44 (integrated adj1 working).tw. 132

45 Quality improvement/ 4716

46 (quality improvement or QI).tw. 2541

47 or/16-46 30212

48 (indicator* or metrics or measurements or measures or indices).ti. 3392

49 7 and 48 290

50 7 and 15 and 47 1799

51 49 or 50 2006

52 (MEDLINE or systematic review).tw. or meta analysis.pt. 4706

53 51 and 52 66

**Web of Science**

#1 TI=(( indicator* OR metrics OR measurements OR measures OR indices )) OR AB=(( indicator* OR metrics OR measurements OR measures OR indices ))

#2 TI=(( primary AND care) OR (primary AND health AND care) OR (primary AND healthcare) OR (general AND practice) OR (gp ))

#3 TS=(( "national general practice improvement programme*" OR gpip OR "national voice*" OR triag* OR "care navigation*" OR "standard actions and outcomes" OR "patient experience*") OR (person-centred AND care) OR ("access to care" OR "telephone access" OR optim?ation OR information-gathering) OR (information AND gathering) OR (expressed AND need*) OR (quality AND improvement) OR (qi ) )

#4 TS=( patient* OR telephone* OR online* OR virtual* NEAR/3 access* OR consult* OR visit* OR appointment* )

#5 TS=( patient* NEAR/3 (access* OR consult* OR visit* OR appointment* ))

#6 TS=( telephone* NEAR/3 (access* OR consult* OR visit* OR appointment* ))

#7 TS=( online* NEAR/3 (access* OR consult* OR visit* OR appointment* ))

#8 TS=( virtual* NEAR/3 (access* OR consult* OR visit* OR appointment* ))

#9 TS=( manag* NEAR/3 workload* )

#10 TS=( "standard actions and outcomes" )

#11 TS= (( time) NEAR/3 sav* )

#12 TS= (( cost) NEAR/3 sav* )

#13 TS= ( staff NEAR/3 ( morale OR condition* OR experience* ))

#14 TS= (( prioritis*) NEAR/2 ( care OR service* OR resource* ))

#15 TS= (( allocat*) NEAR/2 ( care OR service* OR resource* ))

#16 TS=(( multi-professional) NEXT( team* OR framework* ))

#17 TS=((" multi professional") NEXT( team* OR framework* ))

#18 TS=(matching NEAR/5 needs)

#19 TS=((episodic) NEAR/2 (care ))

#20 TS=((continuity) NEAR/2 (care ))

#21 TS=(integrated NEAR/1 working)

#22 #4 OR #5 OR #3 OR #6 OR #7 OR #8 OR #9 OR #10 OR #11 OR #12 OR #13 OR #14 OR #15 OR #16 OR #17 OR #18 OR #19 OR #20 OR #21

#16 #1 AND #2 AND #22

#17 TI= (review OR reviews OR meta-analysis OR meta-analyses)

**Search 2: Primary Research Studies**

**Ovid MEDLINE(R) and Epub Ahead of Print, In-Process, In-Data-Review & Other Non-Indexed Citations, Daily and Versions <1946 to November 27, 2023>**

1 *Primary Health Care/ 55618

2 (primary adj3 care).tw. 175345

3 (pc or pcn*).tw. 111412

4 *General Practice/ 12670

5 "general practice".tw. 38958

6 gp.tw. 49911

7 or/1-6 371836

8 *Benchmarking/ 7327

9 "indicator*".tw. 374122

10 metrics.tw. 72596

11 *"Weights and Measures"/ 1396

12 measurements.tw. 841454

13 measures.tw. 946461

14 indices.tw. 194122

15 Quality Assurance, Health Care/ or *Quality Indicators, Health Care/ 65552

16 or/8-15 2326674

17 "national general practice improvement programme*".tw. 0

18 gpip.tw. 61

19 "national voice*".tw. 40

20 *health services accessibility/ or *access to primary care/ 43046

21 ((patient* or telephone* or online* or virtual*) adj3 (access* or consult* or visit* or appointment*)).tw. 89291

22 *Triage/ 7873

23 "triag*".tw. 28575

24 "care navigation*".tw. 165

25 *Workload/ 9975

26 (manag* adj3 workload*).tw. 826

27 "standard actions and outcomes".tw. 0

28 ((time or cost) adj3 sav*).tw. 43108

29 *"Cost Savings"/ 2249

30 *Patient Satisfaction/ 32185

31 "patient experience*".tw. 24330

32 *patient-centered care/ or *patient navigation/ 14648

33 person-centred care.tw. 1944

34 (staff adj3 (morale or condition* or experience*)).tw. 5176

35 "access to care".kw. 2541

36 "telephone access".kw. 1

37 *"Appointments and Schedules"/ 5000

38 ((prioritis* or allocat*) adj2 (care or service* or resource*)).tw. 22469

39 ((multi-professional or multi professional) adj (team* or framework*)).tw. 454

40 optimi?ation.tw. 205472

41 (information-gathering or information gathering).tw. 1369

42 (matching adj5 needs).tw. 393

43 expressed need*.tw. 560

44 ((episodic or continuity) adj2 care).tw. 10093

45 (integrated adj1 working).tw. 128

46 *Quality Improvement/ 16506

47 (quality improvement or QI).tw. 55848

48 or/17-47 579332

49 (indicator* or metrics or measurements or measures or indices).ti. 218758

50 7 and 49 2780

51 7 and 16 and 48 7278

52 50 or 51 9654

53 (MEDLINE or systematic review).tw. or meta analysis.pt. 447506

54 52 and 53 290

55 exp United Kingdom/ 392185

56 (national health service* or nhs*).ti,ab,in. 279858

57 (english not ((published or publication* or translat* or written or language* or speak* or literature or citation*) adj5 english)).ti,ab. 51424

58 (gb or "g.b." or britain* or (british* not "british columbia") or uk or "u.k." or united kingdom* or (england* not "new england") or northern ireland* or northern irish* or scotland* or scottish* or ((wales or "south wales") not "new south wales") or welsh*).ti,ab,jw,in. 2497005

59 (bath or "bath's" or ((birmingham not alabama*) or ("birmingham's" not alabama*) or bradford or "bradford's" or brighton or "brighton's" or bristol or "bristol's" or carlisle* or "carlisle's" or (cambridge not (massachusetts* or boston* or harvard*)) or ("cambridge's" not (massachusetts* or boston* or harvard*)) or (canterbury not zealand*) or ("canterbury's" not zealand*) or chelmsford or "chelmsford's" or chester or "chester's" or chichester or "chichester's" or coventry or "coventry's" or derby or "derby's" or (durham not (carolina* or nc)) or ("durham's" not (carolina* or nc)) or ely or "ely's" or exeter or "exeter's" or gloucester or "gloucester's" or hereford or "hereford's" or hull or "hull's" or lancaster or "lancaster's" or leeds* or leicester or "leicester's" or (lincoln not nebraska*) or ("lincoln's" not nebraska*) or (liverpool not (new south wales* or nsw)) or ("liverpool's" not (new south wales* or nsw)) or ((london not (ontario* or ont or toronto*)) or ("london's" not (ontario* or ont or toronto*)) or manchester or "manchester's" or (newcastle not (new south wales* or nsw)) or ("newcastle's" not (new south wales* or nsw)) or norwich or "norwich's" or nottingham or "nottingham's" or oxford or "oxford's" or peterborough or "peterborough's" or plymouth or "plymouth's" or portsmouth or "portsmouth's" or preston or "preston's" or ripon or "ripon's" or salford or "salford's" or salisbury or "salisbury's" or sheffield or "sheffield's" or southampton or "southampton's" or st albans or stoke or "stoke's" or sunderland or "sunderland's" or truro or "truro's" or wakefield or "wakefield's" or wells or westminster or "westminster's" or winchester or "winchester's" or wolverhampton or "wolverhampton's" or (worcester not (massachusetts* or boston* or harvard*)) or ("worcester's" not (massachusetts* or boston* or harvard*)) or (york not ("new york*" or ny or ontario* or ont or toronto*)) or ("york's" not ("new york*" or ny or ontario* or ont or toronto*))))).ti,ab,in. 1789355

60 (bangor or "bangor's" or cardiff or "cardiff's" or newport or "newport's" or st asaph or "st asaph's" or st davids or swansea or "swansea's").ti,ab,in. 72207

61 (aberdeen or "aberdeen's" or dundee or "dundee's" or edinburgh or "edinburgh's" or glasgow or "glasgow's" or inverness or (perth not australia*) or ("perth's" not australia*) or stirling or "stirling's").ti,ab,in. 263322

62 (armagh or "armagh's" or belfast or "belfast's" or lisburn or "lisburn's" or londonderry or "londonderry's" or derry or "derry's" or newry or "newry's").ti,ab,in. 34803

63 or/55-62 3133260

64 (exp africa/ or exp americas/ or exp antarctic regions/ or exp arctic regions/ or exp asia/ or exp australia/ or exp oceania/) not (exp United Kingdom/ or europe/) 3368728

65 63 not 64 2964976

66 52 and 65 1668

67 66 not 54 1558

68 limit 67 to yr="2013 -Current" 792

**Embase <1974 to 2023 Week 50>**

1 *primary health care/ 35497

2 (primary adj3 care).tw. 239385

3 (pc or pcn*).tw. 161907

4 *general practice/ 40380

5 "general practice".tw. 45197

6 gp.tw. 75054

7 or/1-6 523769

8 *benchmarking/ 3352

9 "indicator*".tw. 486670

10 metrics.tw. 102184

11 *standard/ 5212

12 measurements.tw. 1011185

13 measures.tw. 1191768

14 indices.tw. 245537

15 health care quality/ 278103

16 *quality control/ 37498

17 or/8-16 3102325

18 "national general practice improvement programme*".tw. 0

19 gpip.tw. 70

20 "national voice*".tw. 51

21 *health care access/ 14139

22 *primary care access/ 42

23 ((patient* or telephone* or online* or virtual*) adj3 (access* or consult* or visit* or appointment*)).tw. 160508

24 *patient triage/ 1401

25 "triag*".tw. 45361

26 "care navigation*".tw. 261

27 *workload/ 12227

28 (manag* adj3 workload*).tw. 1155

29 "standard actions and outcomes".tw. 0

30 ((time or cost) adj3 sav*).tw. 67495

31 *"cost control"/ 9302

32 *patient satisfaction/ 29366

33 "patient experience*".tw. 41909

34 *patient care/ 82314

35 person-centred care.tw. 2371

36 (staff adj3 (morale or condition* or experience*)).tw. 7573

37 "access to care".kw. 2868

38 "telephone access".kw. 1

39 *hospital management/ 28285

40 ((prioritis* or allocat*) adj2 (care or service* or resource*)).tw. 27840

41 ((multi-professional or multi professional) adj (team* or framework*)).tw. 801

42 optimi?ation.tw. 255836

43 (information-gathering or information gathering).tw. 1786

44 (matching adj5 needs).tw. 531

45 expressed need*.tw. 781

46 ((episodic or continuity) adj2 care).tw. 13829

47 (integrated adj1 working).tw. 197

48 *total quality management/ 29882

49 (quality improvement or QI).tw. 91444

50 or/18-49 858351

51 (indicator* or metrics or measurements or measures or indices).ti. 251520

52 7 and 51 3884

53 7 and 17 and 50 12114

54 52 or 53 15433

55 meta-analys:.mp. or search:.tw. or review.pt. 3910626

56 54 and 55 1359

57 limit 56 to (embase or medline or "preprints (unpublished, non-peer reviewed)") 1190

58 exp United Kingdom/ 468873

59 (national health service* or nhs*).ti,ab,in,ad. 482005

60 (english not ((published or publication* or translat* or written or language* or speak* or literature or citation*) adj5 english)).ti,ab. 62063

61 (gb or "g.b." or britain* or (british* not "british columbia") or uk or "u.k." or united kingdom* or (england* not "new england") or northern ireland* or northern irish* or scotland* or scottish* or ((wales or "south wales") not "new south wales") or welsh*).ti,ab,jx,in,ad. 3773444

62 (bath or "bath's" or ((birmingham not alabama*) or ("birmingham's" not alabama*) or bradford or "bradford's" or brighton or "brighton's" or bristol or "bristol's" or carlisle* or "carlisle's" or (cambridge not (massachusetts* or boston* or harvard*)) or ("cambridge's" not (massachusetts* or boston* or harvard*)) or (canterbury not zealand*) or ("canterbury's" not zealand*) or chelmsford or "chelmsford's" or chester or "chester's" or chichester or "chichester's" or coventry or "coventry's" or derby or "derby's" or (durham not (carolina* or nc)) or ("durham's" not (carolina* or nc)) or ely or "ely's" or exeter or "exeter's" or gloucester or "gloucester's" or hereford or "hereford's" or hull or "hull's" or lancaster or "lancaster's" or leeds* or leicester or "leicester's" or (lincoln not nebraska*) or ("lincoln's" not nebraska*) or (liverpool not (new south wales* or nsw)) or ("liverpool's" not (new south wales* or nsw)) or ((london not (ontario* or ont or toronto*)) or ("london's" not (ontario* or ont or toronto*)) or manchester or "manchester's" or (newcastle not (new south wales* or nsw)) or ("newcastle's" not (new south wales* or nsw)) or norwich or "norwich's" or nottingham or "nottingham's" or oxford or "oxford's" or peterborough or "peterborough's" or plymouth or "plymouth's" or portsmouth or "portsmouth's" or preston or "preston's" or ripon or "ripon's" or salford or "salford's" or salisbury or "salisbury's" or sheffield or "sheffield's" or southampton or "southampton's" or st albans or stoke or "stoke's" or sunderland or "sunderland's" or truro or "truro's" or wakefield or "wakefield's" or wells or westminster or "westminster's" or winchester or "winchester's" or wolverhampton or "wolverhampton's" or (worcester not (massachusetts* or boston* or harvard*)) or ("worcester's" not (massachusetts* or boston* or harvard*)) or (york not ("new york*" or ny or ontario* or ont or toronto*)) or ("york's" not ("new york*" or ny or ontario* or ont or toronto*))))).ti,ab,in,ad. 2955454

63 (bangor or "bangor's" or cardiff or "cardiff's" or newport or "newport's" or st asaph or "st asaph's" or st davids or swansea or "swansea's").ti,ab,in,ad. 121659

64 (aberdeen or "aberdeen's" or dundee or "dundee's" or edinburgh or "edinburgh's" or glasgow or "glasgow's" or inverness or (perth not australia*) or ("perth's" not australia*) or stirling or "stirling's").ti,ab,in,ad. 406703

65 (armagh or "armagh's" or belfast or "belfast's" or lisburn or "lisburn's" or londonderry or "londonderry's" or derry or "derry's" or newry or "newry's").ti,ab,in,ad. 56741

66 or/58-65 4613973

67 (exp "arctic and antarctic"/ or exp oceanic regions/ or exp western hemisphere/ or exp africa/ or exp asia/) not (exp united kingdom/ or europe/) 3577298

68 66 not 67 4351606

69 54 and 68 3209

70 69 not 56 2777

71 limit 70 to (embase or medline or "preprints (unpublished, non-peer reviewed)") 2117

72 limit 71 to yr="2013 -Current" 921

**Web of Science**

#1 TI=(( indicator* OR metrics OR measurements OR measures OR indices )) OR AB=(( indicator* OR metrics OR measurements OR measures OR indices ))

#2 TI=(( primary AND care) OR (primary AND health AND care) OR (primary AND healthcare) OR (general AND practice) OR (gp ))

#3 TS=(( "national general practice improvement programme*" OR gpip OR "national voice*" OR triag* OR "care navigation*" OR "standard actions and outcomes" OR "patient experience*") OR (person-centred AND care) OR ("access to care" OR "telephone access" OR optim?ation OR information-gathering) OR (information AND gathering) OR (expressed AND need*) OR (quality AND improvement) OR (qi ) )

#4 TS=( patient* OR telephone* OR online* OR virtual* NEAR/3 access* OR consult* OR visit* OR appointment* )

#5 TS=( patient* NEAR/3 (access* OR consult* OR visit* OR appointment* ))

#6 TS=( telephone* NEAR/3 (access* OR consult* OR visit* OR appointment* ))

#7 TS=( online* NEAR/3 (access* OR consult* OR visit* OR appointment* ))

#8 TS=( virtual* NEAR/3 (access* OR consult* OR visit* OR appointment* ))

#9 TS=( manag* NEAR/3 workload* )

#10 TS=( "standard actions and outcomes" )

#11 TS= (( time) NEAR/3 sav* )

#12 TS= (( cost) NEAR/3 sav* )

#13 TS= ( staff NEAR/3 ( morale OR condition* OR experience* ))

#14 TS= (( prioritis*) NEAR/2 ( care OR service* OR resource* ))

#15 TS= (( allocat*) NEAR/2 ( care OR service* OR resource* ))

#16 TS=(( multi-professional) NEXT( team* OR framework* ))

#17 TS=((" multi professional") NEXT( team* OR framework* ))

#18 TS=(matching NEAR/5 needs)

#19 TS=((episodic) NEAR/2 (care ))

#20 TS=((continuity) NEAR/2 (care ))

#21 TS=(integrated NEAR/1 working)

#22 #4 OR #5 OR #3 OR #6 OR #7 OR #8 OR #9 OR #10 OR #11 OR #12 OR #13 OR #14 OR #15 OR #16 OR #17 OR #18 OR #19 OR #20 OR #21

#23 #1 AND #2 AND #22

#24 TI=(national health service* or nhs*) OR AB=(national health service* or nhs*) OR AD=(national health service* or nhs*) OR OO=(national health service* or nhs*) or OG=(national health service* or nhs*)

#25 TI=(gb or "g.b." or britain* or (british* not "british columbia") or uk or "u.k." or united kingdom* or (england* not "new england") or northern ireland* or northern irish* or scotland* or scottish* or ((wales or "south wales") not "new south wales") or welsh*) OR AB=(gb or "g.b." or britain* or (british* not "british columbia") or uk or "u.k." or united kingdom* or (england* not "new england") or northern ireland* or northern irish* or scotland* or scottish* or ((wales or "south wales") not "new south wales") or welsh*)

#26 #24 OR #25

#27 #23 OR #26

#28 #23 AND #26 and 2024 or 2023 or 2022 or 2021 or 2019 or 2020 or 2018 or 2017 or 2016 or 2015 or 2014 or 2013
