# Supplementary material for: A systematic review of international performance indicators and metrics relevant to UK general practice
Source: BMJ Open Qual. 2025 Oct 15;14(4):e003477. doi: 10.1136/bmjoq-2025-003477 (PMC12530429; doi:10.1136/bmjoq-2025-003477)
Supplement: online supplemental file 2 [file bmjoq-14-4-s002.docx]

**Supplementary file 2: Indicator/metric coverage of included studies**

| **Source** | **Country or region** | **Individual indicators** | | **Indicator groups** | | **Further details of individual indicators available? Yes (specify)/No** |
| --- | --- | --- | --- | --- | --- | --- |
|  |  | Name | GPIP domains covered | Name (n) | GPIP domains covered |  |
| *Umbrella review* |  |  |  |  |  |  |
| Ramalho 2019 | Any | 727 indicators from 33 SRs | Unclear | Process indicators (n = 542) | Unclear | [Yes, see indicator list](https://doi.org/10.1371/journal.pone.0220888.s005) |
|  |  |  |  |  |  |  |
| *Systematic or non-systematic reviews* |  |  |  |  |  |  |
| Brennan 2012 | Any (English language only) | 41 instruments included for full review | 1a; 1b; 5 (as distal outcomes) | Measures of CQI implementation and use; organisational context; and individual factors | 1a; 1b; 5 (as distal outcomes) | Yes (details of development and measurement properties) |
| Brennan 2013 | Any (English language only) | 40 instruments included for full review | 1a; 1b; 5 (as distal outcomes) | Measures of teamwork context; team process and team outcomes | 1a; 1b; 5 (as distal outcomes) | Yes (details of development and measurement properties) |
| Derriennic 2022 | Any (articles in English or French) | 29 instruments included | 5 (patient experience) | N/A | N/A | Yes (details of subscales and measurement properties) |
| Obucina 2018 | Any (English language only) | N/A | N/A | Triple Aim Framework (Experience of Care; Population Health; Cost) | 1b; 2 | No (limited data extracted from included studies) |
| Rendell 2022 | Any (English language only) | Not reported (only indicator type reported) | Unclear but includes primary care quality and performance | Reported as applicable | Unclear but includes primary care quality and performance | No (limited data extracted from included studies) |
| Rhydderch 2005 | Any | Five organisational assessment instruments: Primary Care Assessment Tool (PCAT); Visit in practice (VIP); Multi-method assessment process (MAP); Clinical microsystem survey; Standards and a method for assessing Australian general practices | 1b; 2; 4 (workload and stress mentioned in VIP) | N/A | N/A | Yes (data extracted from included studies) |
| Simou 2015 | Any (English language only) | 556 individual indicators | N/A | 10 indicator groups: OECD Health Care Quality Indicators Project: The expert panel on primary care (27); The 6 European Countries expert panel: Quality indicators for general practice management (62); The European Commission: The European Primary Care Monitoring System (PC Monitor) (99); CIHI: Pan-Canadian Primary Health Care Indicators (105); Agency for Healthcare Research and Quality (AHRQ): National Healthcare Quality Report (NHQR) (31); PPRNet: Accelerating the Translation of Research into Practice (A-TRIP) (54); New Zealand Ministry of Health: PHO Performance Management Program (14); The Australian Primary Health Care Research Institute: National Performance Indicators (11); Royal Australian College of General Practitioners (RACGP): Standards for General Practices (4th edition) (139); The Improvement Foundation (Australia): Australian Primary Care Collaboratives Program (14) | Varies between indicator groups | No |
| Stange 2014 | Any | N/A | N/A | Table of indicator groups (n = unclear) classified by topic | 1a?; 1b; 2; 3; 4; 5 | No (references for indicator groups in table) |
| Yapi 2023 (protocol) | Any (articles in English or French on indicators for interprofessional primary care teams) | N/A (protocol) | N/A | N/A (protocol) | N/A | No |
|  |  |  |  |  |  |  |
| *Primary literature* |  |  |  |  |  |  |
| Alsabbagh 2020 | Canada | Continuity of Care Index (COCI); Usual Provider of Care Index (UPC); Modified Modified Continuity Index (MMCI); Continuity with family physicians; Mental health continuity; Multiple conditions continuity | 2 (continuity of care) | Continuity of care (n = 6) | 2 (continuity of care) | Yes (details of how to calculate each indicator) |
| Benson 2023 | UK (England) | Patient Experience; Result Satisfaction; Service Integration | 5 (improved patient experience) | N/A | N/A | Yes (details of indicators and comparison with three existing measures) |
| Breton 2023 | Canada | Clinician supply; patient demand; third next available appointment; relational continuity; 48-hour capacity; use of walk-in appointments; professional diversity of care; no-shows | 1b; 3 | Primary care dashboard | 1b; 3 | Yes (description of indicators, how calculated and how selected (literature review and expert panel)) |
| Crossland 2014 | Australia | Primary Care Practice Improvement Tool (PC-PIT) | 2 | N/A | N/A | Yes (details of PC-PIT and results of initial validation in six general practices) |
| Engels 2006 | Europe | N/A | N/A | European Practice Assessment instrument (62) | 5 | Yes (number of questions, validity and feasibility) |
| Haj-Ali 2017 | Canada (Ontario) | N/A | N/A | Primary Care Performance Measurement (291; 179 at system level and 112 at practice level) | 1a; 1b; 2; 5 | No |
| Howie 2000 | UK (England and Scotland) | Consultation Quality Index (CQI) based on consultation length, how well patients consider they know the doctor and 'patient enablement' | 5 | N/A | N/A | Yes (details of how CQI was developed and measures of reliability and validity) |
| Kringos 2019 | Europe (European Union) | N/A | N/A | Ten domains of primary care with suggested key indicators for each (universal and accessible; human resources; organisation; integrated; comprehensive and community-oriented; partnership with patients and informal caregivers; co-ordination; continuity; patient-centred; addressing personal health needs) | 2; 5 | Yes (brief list for each domain) |
| Kringos 2010 | Europe (European Union) | N/A | N/A | Primary Care Monitoring System (PC Monitor): Governance (12); Economic conditions (11); Workforce development (16); Access to services (12); Continuity (9); Co-ordination (7); Comprehensiveness (10); Quality (17); Efficiency (5) | 1b; 2; 5 | No |
| Levesque 2012 | Canada | 17 validated instruments with 118 subscales: Components of Primary Care Index (CPCI); Interpersonal Processes of Care (IPC); EUR-OPEP instrument; the General Practice Assessment Questionnaire (GPAQ); Medical Interview Satisfaction Scale (MISS-21); Patient Assessment of Chronic Illness Care (PACIC); Primary Care Assessment Survey (PCAS); Primary Care Assessment Tool (PCAT); Veterans Affairs National Outpatient Satisfaction Survey (VANOCSS); visit-based questionnaires (PEQ, MISS-21, COAHS); Consumer Assessment of Health Plans Study (CAHPS 2.0); Canadian Community Health Survey (CCHS); Patient Experience Questionnaire (PEQ); Patient Satisfaction Questionnaire Short Form (PSQ-18); (Consumer Opinions on Ambulatory Health Services (COAHS) | 1b; 2; 5 | N/A | N/A | No (references provided) |
| Schafer 2013 | Europe | N/A | N/A | GP questionnaire; Patient experience questionnaire; Patient values questionnaire; Practice questionnaire | 1b; 2; 4 | Yes (online supplementary material) |
| Sidaway-Lee 2019 | UK (England) | St Leonard's Index of Continuity of Care (SLICC) | 5 | N/A | N/A | Yes (defined as % of face to face appointments with patient's own personal doctor; data from one general practice over 2 years indicated 51.7% (95% CI 51.2 to 52.2) of appointments were with personal doctor) |
|  |  |  |  |  |  |  |
| *Reports* |  |  |  |  |  |  |
| Dixon 2015 (Kings Fund) | UK (England) | N/A (focus on websites providing multiple indicators) | N/A | NHS Choices (30); MyNHS (48); Primary Care Web Tool (72); PHE National General Practice Profiles (266); CQC Intelligent Monitoring Reports (33); CQC rating of general practices; GP Patient Survey (58); QOF (77) | Varies between indicator groups | Yes (some details in Appendix) |
| IAPO 2012 | International | N/A | N/A | 11 groups of 'system level indicators' | 1b; 2; 5 | Yes (some details in text) |

1. Ramalho A, Castro P, Goncalves-Pinho M, Teixeira J, Santos JV, Viana J, Lobo M, Santos P, Freitas A: **Primary health care quality indicators: An umbrella review**. *PLoS ONE* 2019, **14**(8):e0220888.
