# Supplementary material for: A systematic review of international performance indicators and metrics relevant to UK general practice
Source: BMJ Open Qual. 2025 Oct 15;14(4):e003477. doi: 10.1136/bmjoq-2025-003477 (PMC12530429; doi:10.1136/bmjoq-2025-003477)
Supplement: online supplemental file 4 [file bmjoq-14-4-s004.docx]

**Supplementary file 4: Summary of indicator quality assessments using the AHRQ criteria**

| Reference | Metric/indicator name(s) | Standardisation | Comparability | Availability | Timeliness | Relevance | Validity | Experience | Stability | Evaluability | Distinguishable | Credibility |
| --- | --- | --- | --- | --- | --- | --- | --- | --- | --- | --- | --- | --- |
| Alsabbagh 2020[1] | Primary care performance indicators | Yes (standardised across provinces) | No (doesn’t include data on external factors) | Yes (routinely collected data) | Can’t tell (outside scope of study) | Yes (relevant for decision-makers and patients) | Yes (internal and external validity considered) | Yes | Yes (no evidence metrics are planned to be discontinued) | Partly yes (some indicators clearly evaluable as showing better or worse performance) | Yes (capable of showing differences, though data  not reported in study) | Yes (authors report efforts to assess data conformity,  completeness, and plausibility of results) |
| Benson 2023[2] | Patient experience; Result satisfaction; Service integration | Can’t tell (study measured each parameter in a different way | Can’t tell | Can’t tell | Can’t tell | Can’t tell | Can’t tell | Can’t tell | Can’t tell | Can’t tell | Can’t tell | Can’t tell |
| Breton 2023[3] | Primary health care dashboard | No(cannot be used outside clinic) | No | Yes | Can't tell | Yes(relevant for clinicians in primary health care) | Yes | No(new indicator) | Yes | Yes | Can't tell | Yes |
| Crossland 2014[4] | Primary Care Practice Improvement Tool (PC-PIT) | Can’t tell (indicator under development) | Partly yes (includes practice contextual information) | No (requires practice survey) | Can’t tell (outside scope of study) | Yes (relevant for practice decision-makers) | Partly yes (content validity assessed) | No (new indicator) | Can’t tell (new indicator) | Partly yes (some elements indicate better or worse performance) | Yes (capable of showing differences, though data  not reported in study) | Yes (assessed as useful and relevant by most participants) |
| Engels 2006[5] | European Practice Assessment Instrument | No (different countries had different results) | Yes | Yes | Can't tell | Yes | Partly yes (some indicators were not assessed for validity) | No (new indicator) | Can't tell (new indicator) | Yes | Yes | Yes |
| Haj-Ali 2017[6] | Primary Care Performance Management (PCPM) Framework | No (cannot be used in different settings) | Yes | Yes | Can't tell | Yes (relevant for stakeholders) | Yes | Can't tell | Can't tell | Yes | Yes | Yes |
| Howie 2000[7] | Consultation Quality Index (CQI) | Can’t tell (indicator under development) | Partly yes (includes practice-level deprivation data) | No (requires patient survey) | Can’t tell (indicator under development) | Yes (relevant for assessing quality of GPs’  consultations) | Partly yes (face validity assessed) | No (new indicator) | Can’t tell (new indicator | Partly yes (some elements indicate better or worse performance) | Yes (differences between practices and individuals) | Yes (designed to be part of audit) |
| Kringos 2010[8] | Primary Care Monitoring System (PC Monitor) | Can't tell | Yes | Yes | Can't tell | Yes | Yes | Can't tell | Can't tell(new indicator) | Yes | Yes | Yes |
| Kringos 2019[9] | Not specified but similar to PC Monitor above | Can't tell | Can't tell | Yes | Can't tell | Yes | Yes | Can't tell | Can't tell | Yes | Can't tell | Yes |
| Sidaway-Lee 2019[10] | St Leonard’s Index of Continuity of Care (SLICC) | Can't tell | Yes | Yes | Can't tell | Yes | Yes | Can't tell | Can't tell | Yes | Yes | Yes |

**References for Supplementary file 4**

1. Alsabbagh MW, Kueper JK, Wong ST, Burge F, Johnston S, Peterson S, Lawson B, Chung H, Bennett M, Blackman S *et al*: **Development of comparable algorithms to measure primary care indicators using administrative health data across three Canadian provinces**. *International journal of population data science* 2020, **5**(1):1340.

2. Benson T, Benson A: **Routine measurement of patient experience**. *BMJ open quality* 2023, **12**(1).

3. Breton M, Gaboury I, Bordeleau F, Lamoureux-Lamarche C, Martin E, Deslauriers V, Deville-Stoetzel JB: **Use of Electronic Medical Record Data to Create a Dashboard on Access to Primary Care**. *Healthc Policy* 2023, **18**(4):72-88.

4. Crossland L, Janamian T, Sheehan M, Siskind V, Hepworth J, Jackson CL: **Development and pilot study of the Primary Care Practice Improvement Tool (PC-PIT): an innovative approach**. *Med J Aust* 2014, **201**(3 Suppl):S52-55.

5. Engels Y, Dautzenberg M, Campbell S, Broge B, Boffin N, Marshall M, Elwyn G, Vodopivec-Jamsek V, Gerlach FM, Samuelson M *et al*: **Testing a European set of indicators for the evaluation of the management of primary care practices**. *Family Practice* 2006, **23**(1):137-147.

6. Haj-Ali W, Hutchison B: **Establishing a primary care performance measurement framework for Ontario**. *Healthcare Policy* 2017, **12**(3):66-79.

7. Howie JGR, Heaney DJ, Maxwell M, Walker JJ, Freemana GK: **Developing a 'consultation quality index' (CQI) for use in general practice**. *Family Practice* 2000, **17**(6):455-461.

8. Kringos DS, Boerma WG, Bourgueil Y, Cartier T, Hasvold T, Hutchinson A, Lember M, Oleszczyk M, Pavlic DR, Svab I *et al*: **The European primary care monitor: structure, process and outcome indicators**. *BMC Fam Pract* 2010, **11**:81.

9. Kringos D, Nuti S, Anastasy C, Barry M, Murauskiene L, Siciliani L, De Maeseneer J: **Re-thinking performance assessment for primary care: Opinion of the expert panel on effective ways of investing in health**. *The European journal of general practice* 2019, **25**(1):55-61.

10. Sidaway-Lee K, Gray DP, Evans P: **A method for measuring continuity of care in day-to-day general practice: a quantitative analysis of appointment data**. *Br J Gen Pract* 2019, **69**(682):e356-e362.
