# Supplementary material for: A systematic review of international performance indicators and metrics relevant to UK general practice
Source: BMJ Open Qual. 2025 Oct 15;14(4):e003477. doi: 10.1136/bmjoq-2025-003477 (PMC12530429; doi:10.1136/bmjoq-2025-003477)
Supplement: online supplemental file 5 [file bmjoq-14-4-s005.docx]

**Supplementary file 5: Distribution of indicators across evidence sources**

|  | **Indicators from Ramalho umbrella review[5]** | **Indicators from other studies** | **Indicators used in UK QI studies** |
| --- | --- | --- | --- |
| **Framework domains** | | | |
| 1a: Access to primary care systems/1b: Access to primary care clinical services | Accommodation "patient-focused on": Use of urgent appointments  Cumulative hospitalization days in patients with chronic conditions  Hospital care readmissions in patients with chronic conditions  Urgency care use by patients with chronic conditions  Accommodation "patient-focused on": How early and how late a surgery opened for patient appointment  Accommodation "patient-focused on": Out-of-hours service  Accommodation "patient-focused on": Practice-based triage services  Acceptability: Patient Satisfaction  GPAS questionnaire  Indicator of continuity in the process (index of modified continuity) and of continuity in the outcomes (acute problems, chronic, prevention and psychosocial)  MAAS-global Questionnaire  Accommodation "patient-focused on": Home visits  Percentage of calls able to be handled with telephone advice alone  Availability of telephone triage and advice services  Patient satisfaction of telephone triage and advice services | Distal outcomes of continuous quality improvement (Brennan 2012, 2013)[6, 7]  Triple Aim Framework (Obucina 2018)[9]  Five organisational assessment instruments (Rhydderch 2005)[11]  Indicator groups classified by topic (Stange 2014)[13]  Primary care dashboard (Breton 2023)[17]  Primary Care Performance Measurement (Haj-Ali 2017)[20]  Primary Care Monitoring System (PC Monitor) (Kringos 2010)[23]  17 validated instruments with 118 subscales (Levesque 2012)[24]  Four questionnaires (Schafer 2013)[25]  11 groups of 'system level indicators' (IAPO 2012)[27] | Volume of consultations (Carter)[28]  Online and face-to-face consultations (Carter, Robertson)[28, 30]  Patient-GP contact time (Murdoch)[29]  Appointment duration (Robertson)[30]  Number of calls/day (Slater)[31] |
| 2: Care navigation and triage | Communication centred on the patient  Differentiates appropriately between important and minor issues  Does not raise patient expectations that recommendations will be implemented  Coordination of Care  Patient compliance to advice given to seek emergency care  Patient compliance to advice given to seek GP  Equality in access | Triple Aim Framework (Obucina 2018)[9]  Five organisational assessment instruments (Rhydderch 2005)[11]  Indicator groups classified by topic (Stange 2014)[13]  Continuity of care indicators (Alsabbagh 2020)[15]  Primary Care Practice Improvement Tool (PC-PIT) (Crossland 2014)[18]  Primary Care Performance Measurement (Haj-Ali 2017)[20]  Ten domains of primary care with suggested key indicators for each (Kringos 2019)[22]  Primary Care Monitoring System (PC Monitor) (Kringos 2010)[23]  17 validated instruments with 118 subscales (Levesque 2012)[24]  Four questionnaires (Schafer 2013)[25]  11 groups of 'system level indicators' (IAPO 2012)[27] | Patient surveys (Carter)[28]  Patient satisfaction (Murdoch)[29] |
| 3: Managing demand and capacity | Non-health services  Availability of primary care services  First contact for common health problems  Gatekeeping system  Skill-mix of primary care providers  Utilisation of primary care services | Five organisational assessment instruments (Rhydderch 2005)[11]  Indicator groups classified by topic (Stange 2014)[13]  Primary care dashboard (Breton 2023)[17] | Availability of appointments (Slater)[31]  Non-attendance for pre-booked appointments (Slater)[31] |
| 4: Managing the whole practice workload | Development of the primary care workforce | Indicator groups classified by topic (Stange 2014)[13]  Four questionnaires (Schafer 2013)[25] | Administrative staff workload (Carter, Slater)[28, 31] |
| 5: Key outcome objectives | Up-to-date and confidential medical record keeping  Allocative and productive efficiency  Efficiency in performance of primary care workforce  Employment status of primary care workforce  Primary care practice and team structure  Profile of primary care workforce  Quality management infrastructure in primary care  Recognition and responsibilities | Distal outcomes of continuous quality improvement (Brennan 2012, 2013)[6, 7]  29 validated instruments (Derriennic 2022)[8]  Indicator groups classified by topic (Stange 2014)[13]  Patient Experience; Result Satisfaction; Service Integration (Benson 2023)[16]  European Practice Assessment instrument (Engels 2006)[19]  Primary Care Performance Measurement (Haj-Ali 2017)[20]  Consultation Quality Index (CQI) (Howie 2000)[21]  Ten domains of primary care with suggested key indicators for each (Kringos 2019)[22]  Primary Care Monitoring System (PC Monitor) (Kringos 2010)[23]  17 validated instruments with 118 subscales (Levesque 2012)[24]  St Leonard's Index of Continuity of Care (SLICC) (Sidaway-Lee 2019)[26]  11 groups of 'system level indicators' (IAPO 2012)[27] | Staff surveys |
